# Cost-effectiveness analysis of ropeginterferon alfa-2b for the management of patients with polycythemia vera in Japan

**DOI:** 10.1007/s12185-025-04136-4

**Published:** 2025-12-24

**Authors:** Hiroki Yamaguchi, Yuka Sugimoto, Akihiko Gotoh, Shuichi Ota, Hiromi Igari, Tatsunori Murata, Keigo Hanada, Norio Komatsu, Keita Kirito

**Affiliations:** 1 Department of Hematology, Nippon Medical School, Tokyo, Japan; 2 Department of Hematology and Oncology, Mie University, Mie, Japan; 3 Department of Hematology, Tokyo Medical University, Tokyo, Japan; 4 Department of Hematology, Sapporo Hokuyu Hospital, Hokkaido, Japan; 5 PharmaEssentia Japan KK, Akasaka Center Building 12Th FI, 1-3-13 Motoakasaka, Minato-Ku, Tokyo, 107-0051 Japan; 6 CRECON Medical Assessment Inc, Tokyo, Japan; 7 Departments of Hematology and Advanced Hematology, Juntendo University Graduate School of Medicine, Tokyo, Japan; 8 Department of Hematology and Oncology, University of Yamanashi, Yamanashi, Japan

**Keywords:** Polycythemia vera, Ropeginterferon alfa-2b, Cost-effective, Operational cure, Molecular response

## Abstract

**Objective:**

To verify the clinical benefit of ropeginterferon alfa-2b (ropegIFN) and evaluate its cost-effectiveness compared with hydroxycarbamide or ruxolitinib for patients with polycythemia vera (PV) under the National Health Insurance (NHI) system in Japan.

**Methods:**

The cost-effectiveness of ropegIFN compared with hydroxycarbamide or ruxolitinib was evaluated for patients with PV requiring cytoreductive therapy (without prior cytoreductive therapy) and resistant or intolerant to hydroxycarbamide. According to previous reports, patients with PV who achieve a molecular response with a *JAK*2V617F allele burden < 10% tend to maintain hematologic responses even after discontinuing interferon treatment. Therefore, in these analyses, patients who achieved a molecular response with *JAK*2V617F burden < 10% discontinued ropegIFN treatment.

**Results:**

Compared with hydroxycarbamide, ropegIFN yielded an incremental effectiveness of 0.440 quality-adjusted life years (QALYs), indicating a higher QALY gain. The incremental costs were 128,001,730 yen, and the incremental cost-effectiveness ratio (ICER) was 291,092,030 yen/QALY. Compared with ruxolitinib, the incremental effectiveness of ropegIFN was 1.278 QALYs, while the total costs were reduced by 18,025,182 yen, which resulted in a dominant ICER.

**Conclusions:**

These results suggest that ropegIFN may be cost-effective compared with ruxolitinib in patients with PV who are resistant or intolerant to hydroxycarbamide under the NHI system in Japan.

**Supplementary Information:**

The online version contains supplementary material available at 10.1007/s12185-025-04136-4.

## Introduction

Polycythemia vera (PV) is a myeloproliferative neoplasm caused by aberrations in hematopoietic stem cells and is characterized by an excessive number of red blood cells, which can lead to thrombotic and hemorrhagic events. Furthermore, patients with PV are at risk of developing myelofibrosis or acute myeloid leukemia [1, 2], both of which contribute to a poor prognosis [3, 4]. The current main treatment outcome for patients with PV who receive treatment is the prevention of complications of thrombosis, as stated in JSH Guidelines for Hematological Malignancies (JSH Guidelines), since the prognosis for patients with PV who receive treatment is relatively favorable, with ~ 50% of these patients expected to survive for ≥ 10 years [5]. JSH Guidelines categorize treatment policies according to the risk of patients with PV developing thrombosis and recommend treatment with phlebotomy plus low-dose aspirin in low-risk patients aged < 60 years with no history of thrombosis. In contrast, cytoreductive therapy (e.g., hydroxycarbamide) plus phlebotomy and aspirin therapy is recommended for high-risk patients aged ≥ 60 years or with a history of thrombosis. The use of ruxolitinib is also recommended in patients who are resistant or intolerant to hydroxycarbamide. Following the approval of ropeginterferon alfa-2b (ropegIFN) in Japan in March 2023, JSH Guidelines [6] were revised in 2024 to include ropegIFN as a first-line cytoreductive therapy for high-risk PV patients, as with guidelines in other countries [2, 5, 7].

In the Phase III open-label, randomized-controlled trial (RCT; PROUD-PV study) in Europe of hydroxycarbamide-naïve patients with PV or those currently under treatment, the non-inferiority of ropegIFN to hydroxycarbamide was not demonstrated per the composite primary endpoint of complete hematological response and normal spleen size by imaging at 12 months. However, a Phase IIIb open-label extension study in Europe (CONTINUATION-PV study) has also evaluated the long-term efficacy of ropegIFN compared with best available treatment in patients with PV who enrolled in and completed the PROUD-PV study. Results showed the significant superiority of ropegIFN over best available treatment in terms of “composite outcome including complete hematologic response,” “complete hematological response,” and “molecular response” at 36 months [8]. Further, the high rates of “complete hematologic responses” and “molecular responses" that are reported to persist in patients with PV who receive ropegIFN even after study completion indicate the long-term effectiveness of this treatment [9–11].

The Low-PV study further demonstrated efficacy in patients with low-risk PV [12]. Based on these findings, the revised 2024 JSH Guidelines recommend ropegIFN also for selected low-risk patients with PV, such as those with marked thrombocytosis or splenomegaly. Concerns about secondary malignancies associated with long-term use of hydroxycarbamide or ruxolitinib [13] also support considering ropegIFN in this setting. Taken together, these findings indicate that ropegIFN may be an effective treatment for patients with PV who are under consideration for cytoreductive therapy and are resistant or intolerant to hydroxycarbamide.

In 2023, ropegIFN was designated by the Central Social Insurance Medical Council (Chuikyou) for the Japanese public cost-effectiveness evaluation. The cost-effectiveness evaluation method is stipulated in the Guideline for Preparing Cost-Effectiveness Evaluations to the Central Social Insurance Medical Council (Analytical Guideline) [14]. In this method, quality-adjusted life-year (QALY) obtained by multiplying life years by health state utility (0 [death] to 1 [perfect health]) as an effect measure is used, and cost-effectiveness is evaluated using the incremental cost-effectiveness ratio (ICER), which is a measure that represents the incremental cost needed to acquire 1 QALY. In Japan, Chuikyou will judge the cost-effectiveness to be favorable and maintain the current product price when the ICER for the evaluated product adopted by the Expert Committee conducting the appraisal is < 5 million yen/QALY [15]. The analyses of ropegIFN in the Japanese public cost-effectiveness evaluation system, the results adopted by the Expert Committee were unfavorable in both evaluations: when hydroxycarbamide was used as the comparator for PV patients requiring cytoreductive therapy, without prior cytoreductive therapy, and when ruxolitinib was used as the comparator for PV patients who were resistant or intolerant to hydroxycarbamide.

JSH Guidelines for Japan recommend controlling hematocrit levels in patients with PV who are receiving treatment. Notably, in reports of patients with PV who achieve a molecular response, those with a *JAK2*V617F allele burden < 10% tend to maintain hematologic responses even after discontinuing interferon treatment [5, 10]. Achievement of a molecular response may therefore contribute to treatment discontinuation and a cure. In this context, a cost-effectiveness analysis of ropegIFN for patients with PV taking into account these clinical benefits would help inform healthcare professionals who are considering treatment options for patients with PV. Here, the cost-effectiveness of ropegIFN for patients with PV was re-evaluated using an analysis method that maximizes the expected clinical benefit per the settings of a previous analysis of ropegIFN in the public cost-effectiveness evaluation system in Japan.

## Materials and methods

### Overview

Patients with PV requiring cytoreductive therapy, without prior cytoreductive therapy, and who were resistant or intolerant to hydroxycarbamide were included in the analysis set. Hydroxycarbamide and ruxolitinib were used as comparators to assess the cost-effectiveness of ropegIFN.

A key assumption in these analyses was that patients with PV who achieved a molecular response with *JAK2*V617F burden < 10% would discontinue treatment with ropegIFN [10, 16]. In contrast, discontinuation of ruxolitinib or hydroxycarbamide was not considered owing to a lack of evidence regarding a *JAK2*V617F allele burden < 10% resulting in maintained hematologic responses.

Per the Analytical Guidelines, the analysis was from the perspective of the public healthcare payers, and costs were set as direct medical costs only. Effectiveness measures were assessed using QALY, and the final cost-effectiveness was assessed using ICER. Present values were estimated using a discount rate of 2% per annum for both cost and effects.

### Model structure

**Fig. 1:**
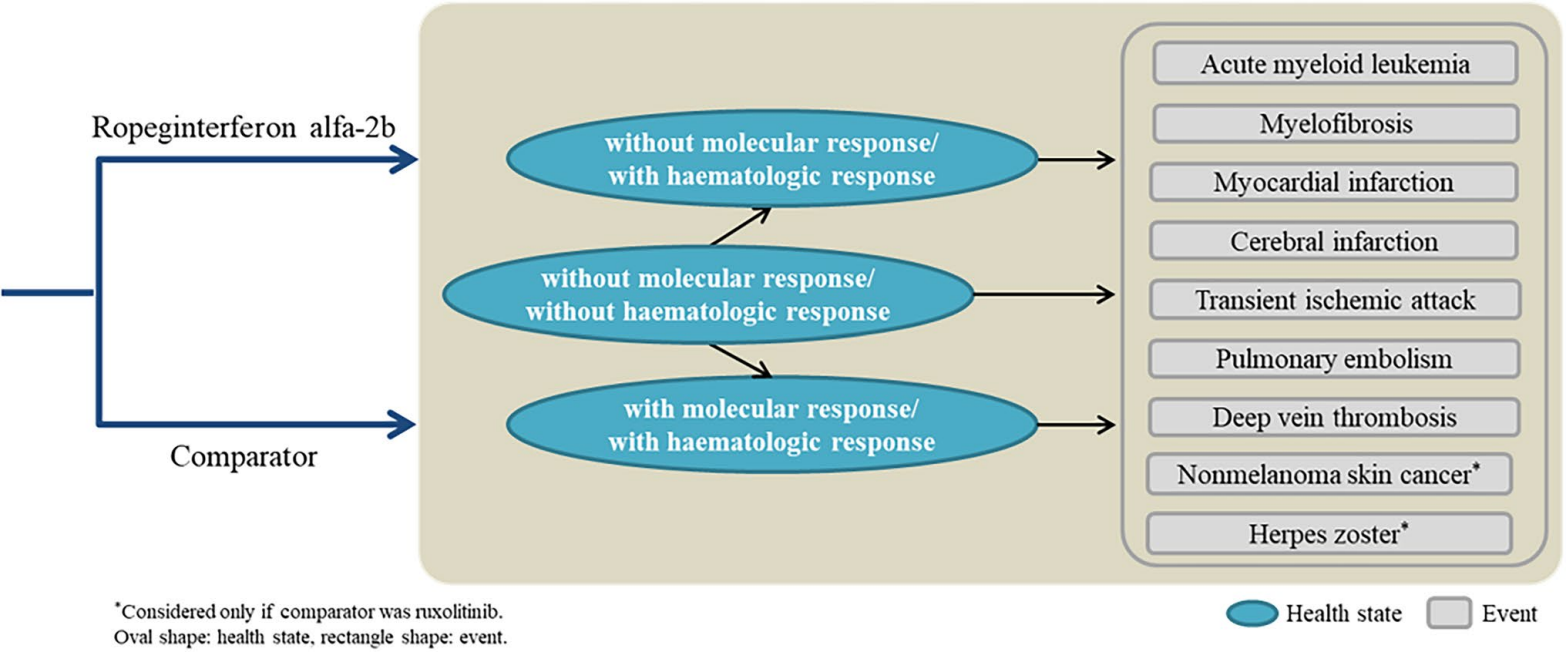
Model structure

In these analyses, an analytical model consisting of four health states was constructed, as follows: “no molecular response/no hematologic response”, “no molecular response/hematologic response”, “molecular response/hematologic response”, and “death” (Fig. 1).

The transition to events associated with PV (acute myeloid leukemia, myelofibrosis, and thrombosis) was also considered for all health states. The following thrombosis-related diseases were included: myocardial infarction, cerebral infarction, transient ischemic attack, pulmonary embolism, and deep vein thrombosis. Analyses using ruxolitinib as the comparator also included non-melanoma skin cancers and herpes zoster, which are associated with this treatment [13, 17].

To simulate the long-term prognosis and morbidity of patients with PV, microsimulations using a Markov model were conducted. These simulations were conducted on 10,000 patients, with an analytical cycle of 1 year and a lifetime time horizon. The analytical model was constructed using Microsoft® Excel® for Microsoft 365 MSO (Version 2202).

### Parameter

#### Clinical parameters

The clinical parameters specified in the analytical model were per the values obtained from previous studies (Table 1).

**Table 1:** List of parameters

Variable	Value	Range for SA [low to high]	Distribution type for PSA	Source
Discount rate per year (cost, effectiveness)	2.00%	0.00–4.00	–	Analysis guideline [14]
Time horizon	50 years	40–60	–	Assumption
Age at start of analysis	58 years	50–64	Normal	Gisslinger et al. [8]
Male ratio	49.5%	39.6–59.4	Beta	Gisslinger et al. [8]
Clinical parameter				
Hematological response rate				
RopegIFN				
At 36 months	70.5%	56.4–84.6	Beta	Gisslinger et al. [8]
Rate ratio of hematological response rate of ropegIFN to hydroxycarbamide				
At 36 months	1.38	1.07–1.79	Log-norm	Gisslinger et al. [8]
Molecular response rate				
RopegIFN				
At 36 months	66.0%	52.8–79.2	Beta	Gisslinger et al. [8]
Rate ratio of molecular response rate of ropegIFN to hydroxycarbamide				
At 36 months	2.31	1.56–3.42	Log-norm	Gisslinger et al. [8]
Proportion of patients whose *JAK2*V617F allele burden decreased to < 10% at 5 years				
RopegIFN	54.3%	43.5–65.2	Beta	Kiladjian et al. [10]
Event incidence				
Thrombosis				
Myocardial infarction	3.1/100 person-year	2.48/100–3.72/100	Beta	Parasuraman et al. [31]
Cerebral infarction	7.6/100 person-year	6.08/100–9.12/100	Beta	
Transient ischemic attack	2.6/100 person-year	2.08/100–3.12/100	Beta	
Pulmonary embolism	2.9/100 person-year	2.32/100–3.48/100	Beta	
Deep vein thrombosis	5.5/100 person-year	4.40/100–6.60/100	Beta	
Hazard ratio for thrombosis in patients without hematologic response (ref: with hematologic response)	1.61	1.03–2.51	Log-norm	
Hematological malignancy				
Acute myeloid leukemia	1.1%/51 months	0.88–1.32	Beta	Dan et al. [18]
Myelofibrosis	2.6%/51 months	2.08–3.12	Beta	
Hazard ratio for acute myeloid leukemia in patients without hematologic response (ref: with hematologic response)	1.22	0.49–3.04	Log-norm	Finazzi et al. [19]
Hazard ratio for myelofibrosis in patients without hematologic response (ref: with hematologic response)	1.84	0.71–4.79	Log-norm	Di et al. [20]
Other event				
Nonmelanoma skin cancer	72.12/1000 person-year	45.72/1000–108.21/1000	Beta	Lin et al. [13]
Herpes zoster	5.3/100 person-year	4.24/1000–6.36/1000	Beta	Verstovsek et al. [21]
Hazard ratio for nonmelanoma skin cancer in patients with ruxolitinib exposure (ref: without ruxolitinib exposure)	2.69	1.03–7.02	Log-norm	Lin et al. [13]
Odds ratio for herpes zoster in patients with ruxolitinib exposure (ref: without ruxolitinib exposure)	7.39	1.33–41.07	Log-norm	Luo et al. [17]
Mortality				
Sex- and age-specific mortality rate	–	–	–	Abridged Life Tables [23]
Thrombosis				
Myocardial infarction				Nomura et al. [32]
First month after event onset	5.0%/month	0.0–5.0	Beta	
After 1 month	0.98%/year	0.93–1.74	Beta	
Cerebral infarction				
First month after event onset	12.9%/month	9.7–16.1	Beta	
After 1 month	1.29%/year	1.03–1.55	Beta	
Pulmonary embolism	9.4%	7.52–11.28	Beta	Kamae et al. [33]
Deep vein thrombosis	9.4%	7.52–11.28	Beta	
Hematological malignancy				
Acute myeloid leukemia	26%/13 weeks	20.8–31.2	Beta	Hjelmgren et al. [34]
Myelofibrosis	2.1%/13 weeks	1.68–2.52	Beta	
Other event				
Nonmelanoma skin cancer	1.1%/year	0.89–1.33	Beta	Cancer Information Service [35]
Health state utility				
Health state utility of patients with PV				
Without molecular response/without hematological response	0.776	0.741–0.811	Beta	Health state utility estimation using a TTO method
Incremental utility in the hematologic response	± 0.000	–	Beta	
Incremental utility in the molecular response	+ 0.034	0.008–0.060	Beta	
Disutility with ruxolitinib exposure	–0.071	–0.101 to –0.041	Beta	
Disutility at event occurrence				
Thrombosis				
Myocardial infarction	–0.073	–0.088 to –0.058	Beta	Shiroiwa et al. [36]
Cerebral infarction	–0.265	–0.318 to –0.212	Beta	
Transient ischemic attack	–0.032	–0.080 to 0.000	Beta	Sullivan et al. [37]
Pulmonary embolism	–0.051	–0.076 to –0.026	Beta	
Deep vein thrombosis	–0.051	–0.076 to –0.026	Beta	
Hematological malignancy				
Acute myeloid leukemia	–0.037	–0.085 to 0.000	Beta	Sullivan et al. [37]
Myelofibrosis	–0.037	–0.085 to 0.000	Beta	
Other event				
Nonmelanoma skin cancer	–0.002	–0.015 to 0.000	Beta	Sullivan et al. [37]
Herpes zoster	–0.014	–0.017 to –0.011	Beta	Shiragami et al. [38]
Cost				
Treatment cost				
RopegIFN				
First month after treatment start	592,633 yen/month	474,107–711,160	Gamma	Expert opinion
Before molecular response	1,126,722 yen/month	901,378–1,352,067	Gamma	
After molecular response	1,126,722 yen/month	901,378–1,352,067	Gamma	
Hydroxycarbamide				
First month after treatment start	12,683 yen/month	10,146–15,219	Gamma	Expert opinion
Before molecular response	11,537 yen/month	9,230–13,844	Gamma	
After molecular response	11,537 yen/month	9,230–13,844	Gamma	
Ruxolitinib				
First month after treatment start	746,315 yen/month	597,052–895,578	Gamma	Expert opinion
Before molecular response	746,315 yen/month	597,052–895,578	Gamma	
After molecular response	746,315 yen/month	597,052–895,578	Gamma	
Management cost				
RopegIFN: patients with PV requiring cytoreductive therapy without prior cytoreductive therapy				
First month after treatment start	97,477 yen/month	77,982–116,972	Gamma	Expert opinion
Before molecular response	15,118 yen/month	12,095–18,142	Gamma	
After molecular response	15,003 yen/month	12,002–18,003	Gamma	
RopegIFN: patients with PV who were resistant or intolerant to hydroxycarbamide				
First month after treatment start	81,155 yen/month	64,924–97,386	Gamma	Expert opinion
Before molecular response	13,569 yen/month	10,855–16,283	Gamma	
After molecular response	13,569 yen/month	10,855–16,283	Gamma	
Hydroxycarbamide				
First month after treatment start	87,048 yen/month	69,639–104,458	Gamma	Expert opinion
Before molecular response	9,749 yen/month	7,799–11,699	Gamma	
After molecular response	9,749 yen/month	7,799–11,699	Gamma	
Ruxolitinib				
First month after treatment start	80,356 yen/month	64,285–96,427	Gamma	Expert opinion
Before molecular response	11,671 yen/month	9,336–14,005	Gamma	
After molecular response	10,860 yen/month	8,688–13,032	Gamma	
Event treatment cost				
Thrombosis				
Myocardial infarction				Nomura et al. [32]
First month after event onset	2,156,290 yen/month	1,617,218–2,695,362	Gamma	
After 1 month	495,600 yen/12 months	459,996–570,000	Gamma	
Cerebral infarction				
First month after event onset	1,440,107 yen/month	1,080,080–1,800,134	Gamma	
After 1 month	318,387 yen/12 months	262,992–372,996	Gamma	
Transient ischemic attack	472,088 yen	377,670–566,506	Gamma	Kaku et al. [39]
Pulmonary embolism	1,123,451 yen	898,761–1,348,141	Gamma	Kamae et al. [33]
Deep vein thrombosis	1,123,451 yen	898,761–1,348,141	Gamma	
Hematological malignancy				
Acute myeloid leukemia	2,724,420 yen	2,179,536–3,269,304	Gamma	Expert opinion
Myelofibrosis	1,441,099 yen	1,152,879–1,729,319	Gamma	
Other event				
Nonmelanoma skin cancer	131,070 yen	104,856–157,284	Gamma	Japanese DPC
Herpes zoster	34,664 yen	27,731–41,597	Gamma	Imafuku et al. [40]

DPC, diagnosis procedure combination; PSA, probabilistic sensitivity analysis; PV, polycythemia vera; ref, reference; ropegIFN, ropeginterferon alfa-2b; SA, sensitivity analysis; TTO, time trade-off

When patients with PV requiring cytoreductive therapy without prior cytoreductive therapy were analyzed, the response rates at 36 months in the ropegIFN arm of the CONTINUATION-PV study were cited for hematologic and molecular response rates. Hematologic and molecular response rates compared with hydroxycarbamide were established by adjusting response rates with ropegIFN using rate ratios in the control arm of the CONTINUATION-PV study [8]. When patients with PV who were resistant or intolerant to hydroxycarbamide were analyzed, no studies were identified that directly compared ruxolitinib with ropegIFN. Indirect comparisons for efficacy outcomes (hematologic response and molecular response) were considered unfeasible due to the non-negligible heterogeneity in the background characteristics of patients in each clinical study. To help ensure a conservative analysis of ropegIFN, the response rates with ruxolitinib and ropegIFN were assumed to be similar. In addition, patients who achieved a molecular response were assumed to have also achieved a hematologic response, and that by continuing PV treatment from 36 months onwards, their response rates would be maintained for all treatments.

For the proportion of patients attaining a *JAK2*V617F allele burden < 10% with ropegIFN, the values reported at 5 years in the CONTINUATION-PV study were cited. Because patients who achieved a molecular response and attained a *JAK2*V617F allele burden < 10% tended to maintain hematologic responses even after discontinuing interferon treatment, those who were discontinued from ropegIFN treatment were analyzed on the assumption that their respective response rates at 5 years were maintained [10].

Previous study cited the incidence rates of hematologic malignancies did not report incidence of hematologic malignancies according to whether or not patients achieved hematologic responses. The incidence of hematologic malignancies in patients who attained a hematologic response was therefore adjusted using the hazard ratio for the incidence of hematologic malignancies in the absence of a hematologic response reported in other studies [18–20]. Because no previous studies have evaluated the effect on the incidence of thrombosis or hematologic malignancies in patients with a molecular response, the incidence of any event was determined to vary according to the presence or absence of hematologic responses in these analyses.

For the analysis with ruxolitinib as the comparator, the incidence of non-melanoma skin cancer and herpes zoster with ruxolitinib from observational studies was utilized [13, 21]. Non-melanoma skin cancer and herpes zoster were not reported as adverse events related to the ropegIFN arm in the PROUD-PV and CONTINUATION-PV studies. The incidence for each event when ropegIFN was used was therefore set by adjusting the incidence of non-melanoma skin cancer and herpes zoster with ruxolitinib using hazard ratios for ruxolitinib reported in other studies [13, 17].

Given that patients with PV are expected to have a life expectancy equivalent to that of the general population when appropriate treatment is administered as a reference [22], the age–sex-specific general mortality rate in Japanese individuals was set as the mortality rate of patients with PV [23]. In addition, the age–sex-specific general mortality rate was adopted within the simulation of the analytic model when this mortality rate exceeded that at each onset of events.

#### Health state utility parameters

In these analyses, health state utility was set to values estimated from the health state utility estimation using the time trade-off (TTO) method in the general population, and utility at the onset of events was set to values obtained per the previous studies (Table 1).

The utility for each health state was set per a health state utility estimation using the TTO method conducted in 208 patients from the general population in Japan to assess the health economic value of potential future treatment discontinuation or operational cure associated with a molecular response in patients with PV (Supplementary 1). A vignette was developed consisting of the presence or absence of concerns about second malignancies and expectations regarding treatment discontinuation and operational cure per previous studies and the expert clinical opinions of the authors, who are specialists in the treatment of patients with PV. For “no molecular response/no hematologic response,” the health state utility estimated from the “improvement of PV symptoms/no anxiety of second malignancies/no expectation of treatment discontinuation or operational cure” vignette was set. The mean change in health state utility after achieving a molecular response was set as the mean intra-individual change in participants estimated from the vignettes of “improvement of PV symptoms, no anxiety of second malignancy, expectation of treatment discontinuation or operational cure” and “improvement of PV symptoms, no anxiety of second malignancy, no expectation of treatment discontinuation or operational cure”. The incremental health state utility per EuroQOL 5 dimension 3-level (EQ-5D-3L) in the CONTINUATION-PV study when hematologic responses were achieved did not differ between arms regarding the changes from baseline. Therefore, the health state utility was not adjusted to help ensure a conservative analysis for ropegIFN in cases where only hematologic responses were achieved. In these analyses, the effects of anxiety of second malignancies on health state utility when ruxolitinib is used were taken into account, and the change in health state utility when ruxolitinib is used was set as the mean intra-individual change in survey participants as estimated from the vignettes of “improvement of PV symptoms, anxiety of second malignancies, no expectation of treatment discontinuation or operational cure” and “improvement of PV symptoms, no anxiety of second malignancy, no expectation of treatment discontinuation or operational cure”.

#### Cost parameters

In these analyses, the costs of medications for patients with PV, the management costs of administering PV therapy (including hospitalization, outpatient consultation, and laboratory testing costs), and the cost of treatment at the onset of acute myeloid leukemia or myelofibrosis were determined from the opinions of clinical experts. The costs of treatment at the onset of thrombosis-related events were set according to the values obtained from previous studies (Table 1).

The costs of medications for patients with PV, management costs, and treatment costs at the onset of acute myeloid leukemia or myelofibrosis were calculated via micro-costing (e.g., by medical fee schedule), per the definitions of the standard clinical process established from the opinions of clinical experts. The medical fee schedule and drug price list as of March 2025 were used to calculate the costs.

In these analyses, the patients who achieved a molecular response at 5 years and attained a *JAK2*V617F allele burden < 10% were assumed to be discontinued from ropegIFN treatment, such that there would be no costs for ropegIFN from 5 years on for patients who discontinued [10].

### Sensitivity analysis

A deterministic sensitivity analysis was conducted to evaluate the effects of each parameter on the analysis result, and a tornado diagram was generated. The range of variation for parameters was 0–4% for the discount rate, and when no statistical information on the 95% confidence interval was reported in the citation source for other parameters, the range of variation was set as ± 20% of the base set value. A probabilistic sensitivity analysis with 1000 Monte Carlo simulations was conducted to assess the uncertainty of the analysis result. The probability distribution of each parameter was assumed to be gamma distribution for cost parameters, beta distribution for probability parameters and health state utility parameters, and log-normal distribution for proportion parameters [24]. When statistical data on the variation of each parameter were not available, a theoretical distribution was established using 10% of the base set value as the standard error. In addition, a scenario analysis was conducted, where the setting was changed to hydroxycarbamide treatment that did not result in a molecular response.

## Results

### Base-case analysis

When patients with PV requiring cytoreductive therapy without prior cytoreductive therapy were analyzed, the expected QALY for ropegIFN was 9.311 QALY and for hydroxycarbamide was 8.871 QALY, and the incremental effectiveness for ropegIFN was 0.440 QALY. The total costs of treatment were 144,733,280 yen for ropegIFN and 16,731,551 yen for hydroxycarbamide; the incremental cost for ropegIFN was 128,001,730 yen. On analysis of patients with PV requiring cytoreductive therapy without prior cytoreductive therapy, the ICER for ropegIFN when compared with hydroxycarbamide was 291,092,030 yen/QALY.

On analysis of patients with PV who were resistant or intolerant to hydroxycarbamide, the expected QALY for ropegIFN was 9.272 QALY and for ruxolitinib was 7.994 QALY; the incremental effectiveness for ropegIFN was 1.278 QALY. The total costs of treatment were 144,465,437 yen for ropegIFN and 162,490,619 yen for ruxolitinib; the total cost was reduced by 18,025,182 yen due to ropegIFN. On analysis of patients with PV who were resistant or intolerant to hydroxycarbamide, the ICER for ropegIFN compared with ruxolitinib was thus dominant (Table 2).

**Table 2:** Base-case analysis

	Total cost (yen)	Incremental cost (yen)	Effectiveness (QALY)	Incremental effectiveness (QALY)	ICER (yen/QALY)
Patients with PV requiring cytoreductive therapy without prior cytoreductive therapy					
RopegIFN	144,733,280	128,001,730	9.311	0.440	291,092,030
Hydroxycarbamide	16,731,551		8.871		
Patients with PV who were resistant or intolerant to hydroxycarbamide					
RopegIFN	144,465,437	–18,025,182	9.272	1.278	Dominant
Ruxolitinib	162,490,619		7.994		

ICER, incremental cost-effectiveness ratio; PV, polycythemia vera; QALY, quality-adjusted life years; ropegIFN, ropeginterferon alfa-2b

### Sensitivity analysis

**Fig. 2:**
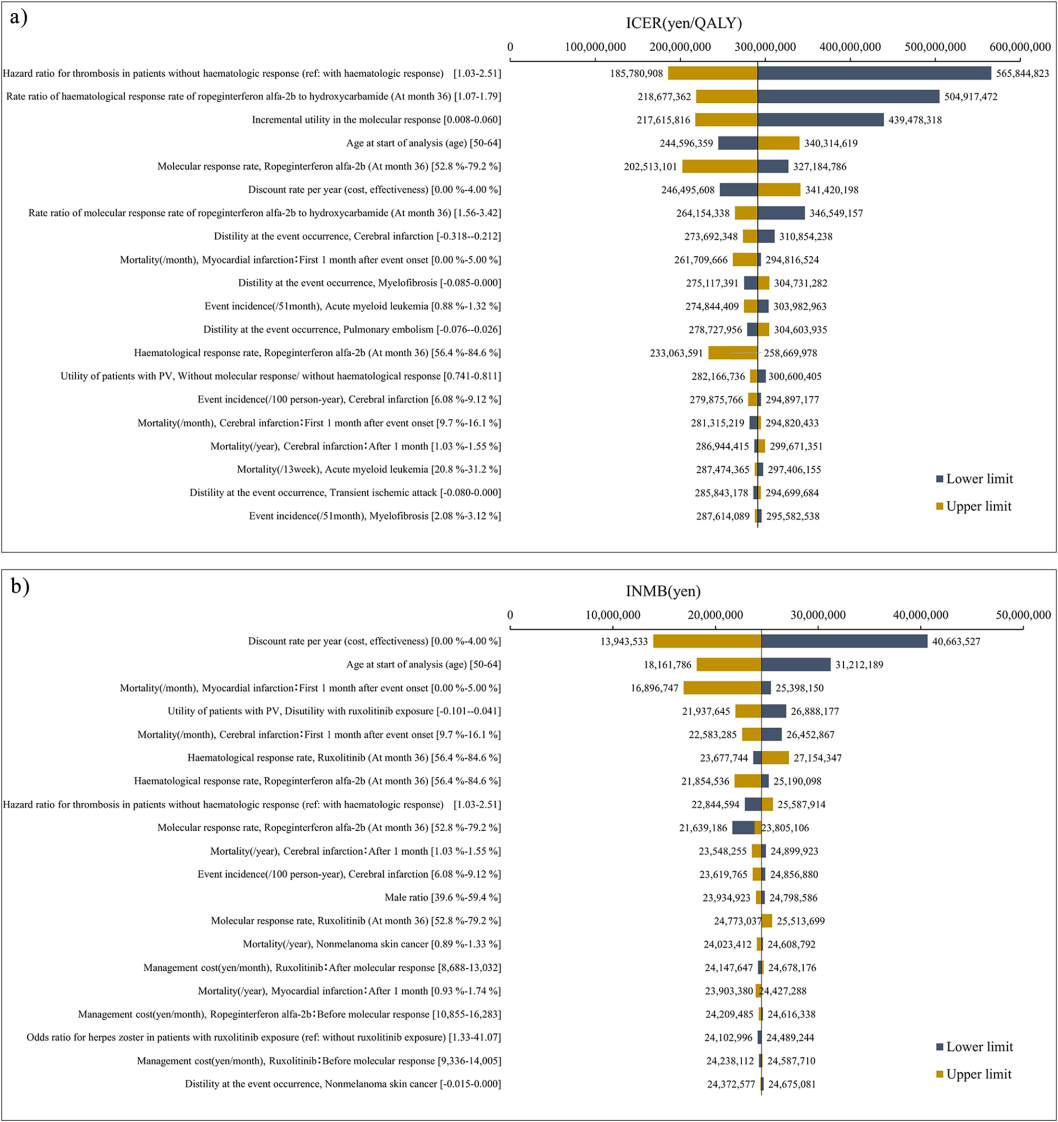
One-way sensitivity analysis of **a** patients with PV requiring cytoreductive therapy without prior cytoreductive therapy and **b** patients with PV who were resistant or intolerant to hydroxycarbamide. (a) ICER, incremental cost-effectiveness ratio; PV, polycythemia vera; QALY, quality-adjusted life years. (b) INMB, incremental net monetary benefit; PV, polycythemia vera

The deterministic sensitivity analysis in patients with PV requiring cytoreductive therapy without prior cytoreductive therapy showed that—among the parameters set in the analytical model—the parameter that most affected the analysis result was the hazard ratio for the incidence of thrombosis in patients without hematologic response. Instead of ICER, incremental net monetary benefit (INMB) was used in the deterministic sensitivity analysis of patients with PV who were resistant or intolerant to hydroxycarbamide, as the base-case analysis result was dominant. INMB was calculated using the following formula:

$$\mathrm{INMB}=\left\{\text{incremental effectiveness }\left(\mathrm{QALY}\right)\text{ x ICER reference value }\left(\frac{5\text{ million yen}}{\mathrm{QALY}}\right)\right\}-\text{incremental cost},$$

where the deterministic sensitivity analysis in patients with PV who were resistant or intolerant to hydroxycarbamide showed that—among the parameters set in the analytical model—the parameter that most affected the analysis result was the discount rate (Fig. 2).

**Fig. 3:**
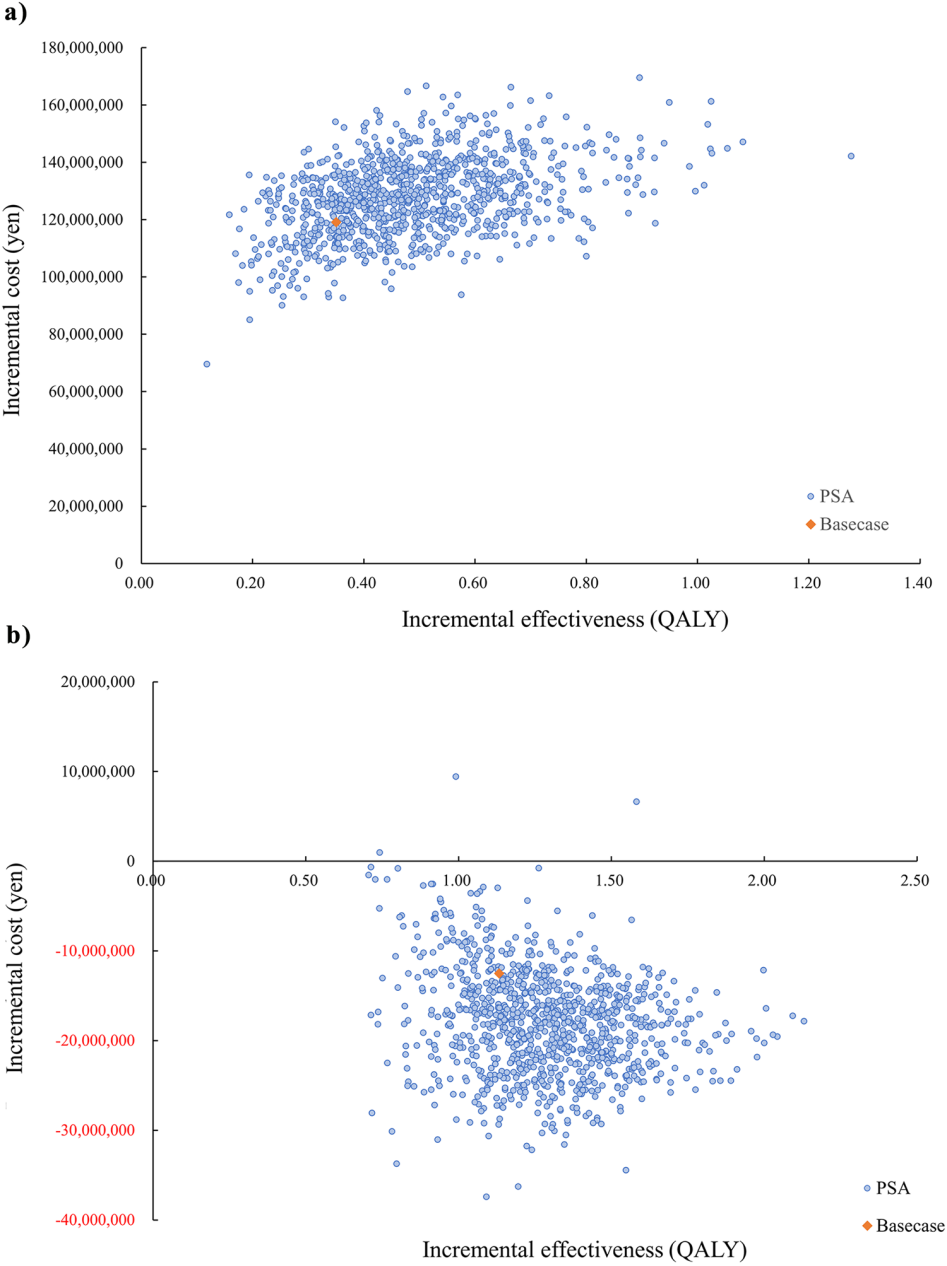
Probabilistic sensitivity analysis of **a** patients with PV requiring cytoreductive therapy without prior cytoreductive therapy and **b** patients with PV who were resistant or intolerant to hydroxycarbamide. (a). ICER, incremental cost-effectiveness ratio; PV, polycythemia vera; PSA, probabilistic Sensitivity Analysis, QALY, quality-adjusted life years. (b) INMB, incremental net monetary benefit; PV, polycythemia vera; PSA, probabilistic Sensitivity Analysis, QALY, quality-adjusted life years

The probabilistic sensitivity analysis in patients with PV requiring cytoreductive therapy without prior cytoreductive therapy showed that the probability of ropegIFN being deemed cost-effective was 0% when the ICER reference value were set to 5 million yen/QALY, 7.5 million yen/QALY, or 10 million yen/QALY. The probabilistic sensitivity analysis in patients with PV who were resistant or intolerant to hydroxycarbamide showed that the probability of ropegIFN being deemed cost-effective for each ICER reference value was as follows: 5 million yen/QALY, 99.9%; 7.5 million yen/QALY, 99.9%; and 10 million yen/QALY, 100.0% (Fig. 3).

The ICER for ropegIFN compared with hydroxycarbamide was 215,640,204 yen/QALY following a scenario analysis wherein the setting was changed to treatment with hydroxycarbamide that did not result in a molecular response.

## Discussion

Building upon a previous analysis from the public cost-effectiveness evaluation system in Japan, the present analyses re-evaluated the cost-effectiveness of ropegIFN using a method designed to maximize its expected clinical benefits. The present analyses in patients with PV requiring cytoreductive therapy without prior cytoreductive therapy demonstrated that the ICER for ropegIFN compared with hydroxycarbamide was 291,092,030 yen/QALY. The ICER was dominant for ropegIFN compared with ruxolitinib in patients with PV who were resistant or intolerant to hydroxycarbamide.

In the present analyses, to maximize the expected clinical benefits of treatment with ropegIFN, the following points were optimized per the analytical settings adopted by the previous analysis of ropegIFN in the public cost-effectiveness evaluation system in Japan. First, patients who achieved a molecular response with a *JAK2*V617F allele burden < 10% tend to maintain a hematologic response even after treatment discontinuation [10]. In addition, the definition of operational cure in the 2021 ELN recommendation (*JAK2*V617F allele burden < 10%, hematological response maintained for ≥ 2 years, no disease progression, onset of thromboembolic events, or worsening of symptoms) was per the PROUD/CONTINUATION-PV studies [16]. A setting was therefore added to discontinue treatment with ropegIFN when patients attained a *JAK2*V617F allele burden < 10%. Second, for the analysis with ruxolitinib as the comparator, the events reported to be associated with ruxolitinib of non-melanoma skin cancers and herpes zoster were added [13, 17]. Third, treatment with ropegIFN may have psychological effects associated with potential treatment discontinuation or operational cure, and may thus reduce anxiety regarding second malignancies, which have been noted with current PV treatments. The improvement in health state utility was therefore attributed to molecular response and reduced anxiety regarding second malignancies, as estimated by a health state utility estimation via the TTO method in a survey of the general population in Japan.

The main changes from the analytical setting adopted by the Expert Committee in the population of patients with PV requiring cytoreductive therapy without prior cytoreductive therapy were setting the discontinuation of ropegIFN treatment, and the setting for health state utility. RopegIFN is reportedly more effective than other PV treatments, as evidenced by attainment of a molecular response and a higher proportion of patients with a *JAK2*V617F allele burden < 10% [8]. In the present analysis, the ICER for ropegIFN was > 5 million yen/QALY, despite the incremental effectiveness obtained from molecular responses being higher and the incremental cost being lower due to early discontinuation than the results adopted by the Expert Committee. As there were no previous studies evaluating the impact on the incidence of thrombosis or hematologic malignancies in patients with a molecular response, the incidence of any event was determined to vary according to the presence or absence of hematologic responses in these analyses. However, it has been reported that the molecular response is associated with a reduced risk of each event [25]. Given that treatment for patients with PV is lifelong, the expectation is that the ICER for ropegIFN will improve when the following points become clearer: the incidence rate of each event in patients with molecular response, the relationship between maintenance of the state of remission with ropegIFN, the possibility of discontinuing treatment, and health state utility.

The main adaptions per the analytical setting adopted by the Expert Committee in the population of patients with PV who were resistant or intolerant to hydroxycarbamide were as follows: setting the discontinuation of ropegIFN treatment, the setting of events reported to be associated with ruxolitinib, and the setting for health state utility. Although the price for ropegIFN is higher than that for ruxolitinib, the total cost is lower than that for ruxolitinib when considering the discontinuation of treatment in patients with PV who receive ropegIFN and achieve a molecular response. Considering the anxiety of second malignancies and events of non-melanoma skin cancer and herpes zoster, the incremental effectiveness of ropegIFN was higher than that when hydroxycarbamide was used as the comparator. As this resulted in the ICER for ropegIFN compared with ruxolitinib becoming dominant, the price for ropegIFN increased and a price threshold exceeding the ICER reference value of 5 million yen/QALY in the public cost-effectiveness of Japan evaluation system was confirmed. The results of the threshold analysis showed that the ICER for ropegIFN compared with ruxolitinib was > 5 million yen/QALY when the price of ropegIFN increased by more than 18.7% from that in March 2025 (Supplementary 2). In Japan, the price is set for each medication and does not change per the population to whom the medication is administered or the indication. However, even when the price for ropegIFN is increased by 18.7%, it may be a cost-effective treatment for patients with PV who are resistant or intolerant to hydroxycarbamide. In the absence of studies directly comparing ruxolitinib with ropegIFN and to keep the present analyses conservative for ropegIFN, the hematologic and molecular response rates for ruxolitinib and ropegIFN were considered the same as those for ruxolitinib and ropegIFN. Further comparisons of the efficacy of ropegIFN and ruxolitinib and long-term safety results are required to provide cost-effective analyses that are more clinically reflective.

The results of the present analyses are consistent with studies that evaluated the cost-effectiveness of ropegIFN in patients with PV in the United States and Austria [26, 27]. The study in the United States assessed the cost-effectiveness of replacing first- or second-line therapy in patients with low- or high-risk PV with ropegIFN from the perspective of the United States healthcare system (the comparator was the existing treatment strategy of using hydroxycarbamide as first-line therapy and ruxolitinib as second-line therapy) [26]. Results showed that the ICER for ropegIFN compared with the existing treatment strategy was USD 141,783/QALY, which was below the standard willingness to pay the threshold of USD 150,000/QALY; thus, ropegIFN was reported as a cost-effective treatment option. This finding that ropegIFN was more cost-effective even without considering the discontinuation of ropegIFN, may be attributed to the annual cost of ruxolitinib being set higher than that of ropegIFN. In contrast, the cost-effectiveness analysis of ropegIFN conducted in Austria evaluated that of ropegIFN plus phlebotomy compared with phlebotomy alone in patients with low-risk PV from the perspective of the Austrian healthcare system [27]. Results showed that the ICER for ropegIFN plus phlebotomy compared with phlebotomy alone was EUR 35,525/QALY. The ICER for ropegIFN was lower than the threshold set per the GDP of Austria (EUR 52,372), making it a cost-effective treatment option. RopegIFN was more cost-effective, even when phlebotomy, which is less expensive than ropegIFN, was used as the comparator. This result may be attributed to permissibility for switching to hydroxycarbamide from phlebotomy therapy and switching to other treatments (high-dose interferon, hydroxycarbamide, and ruxolitinib) also being considered for ropegIFN, which reduced differences in cost. In addition, the early use of ropegIFN may have helped reduce the transition to events associated with high treatment costs and high levels of health state disutility. Although simple comparisons between these studies and the present analyses are difficult given differences in the analytical model structure, settings, and clinical environment, all studies reported that ropegIFN is more cost-effective than other available treatments.

The present study includes several limitations that should be considered when interpreting results. First, given the lack of studies evaluating the efficacy of ropegIFN compared with other medications in patients with PV in Japan, the efficacy parameters used in the analytical modeling of the present study are per those of studies in other countries. No studies were identified that assessed the efficacy of ropegIFN in patients with PV who were refractory to hydroxycarbamide, including the PROUD-PV and CONTINUATION-PV studies that were cited for parameters of ropegIFN efficacy. The present study could therefore not include data from this patient population. Although validation that accounts for the relapse of patients with PV and resumption after discontinuation of ropegIFN is warranted, relapses were not considered in the analytical modeling of the present study owing to the lack of real-world evidence. Further, because evidence of an association with "operational cure" could not be identified, it was assumed for the purpose of the present study that treatment other than ropegIFN would be continued. Additionally, a withdrawal syndrome has been reported for ruxolitinib in patients with myelofibrosis [28, 29]. The cost parameters and analytical settings that could not be obtained from the previous studies were set per the opinions of clinical experts; however, these may not fully reflect the treatment provided to patients with PV in Japan. Although the health state utility estimation using the vignette-based TTO method was set for the present analyses, the vignettes cannot account for all manifestations or conditions of patients with PV. Similarly, the sample size was set to be adequate for estimations of health state utility per previous studies using the TTO method; however, they may not fully represent the general population in Japan.

Under the NHI system in Japan, treatment strategies for patients with PV without a history of prior cytoreductive therapies requiring cytoreductive therapy need to be considered in terms of efficacy, safety, and cost-effectiveness. RopegIFN may also be a more cost-effective treatment option compared with ruxolitinib in patients with PV who are resistant or intolerant to hydroxycarbamide. Owing to the aforementioned limitations, the present analyses were conducted per several hypotheses. Although a recent case report of patients with PV from Japan described treatment discontinuation with ropegIFN, the available evidence remains limited [30]. The cost-effectiveness of ropegIFN for Japanese patients with PV is expected to be re-evaluated following the accumulation of data from these patients, from direct comparisons of ropegIFN with ruxolitinib, and regarding an operational cure.

## Supplementary Information

Below is the link to the electronic supplementary material.

Supplementary file1 (PDF 358 KB)

## Data Availability

The datasets used in this study are available from the corresponding author on reasonable request.
