# Supplementary material for: Cost-effectiveness analysis of ropeginterferon alfa-2b for the management of patients with polycythemia vera in Japan
Source: Int J Hematol. 2025 Dec 24;123(4):556–69. doi: 10.1007/s12185-025-04136-4 (PMC13083320; doi:10.1007/s12185-025-04136-4)
Supplement: Supplementary file 1 — Supplementary file1 (PDF 358 KB) [file 12185_2025_4136_MOESM1_ESM.pdf]

Supplementary 1. Overview of health state utility estimations via the time trade-off method

Objective

To estimate the impact of the psychological effects of potential future treatment discontinuation or operational cure associated with a molecular response, and reducing anxiety of second malignancies associated with long-term treatment, which is a concern for pre-existing polycythemia vera medications, on the health state utility (quality of life score) of patients with polycythemia vera (PV).

Methods

Study design

Health state utility used in health economic evaluations must reflect those of the general population. For this reason, health state utility for each health state were estimated using an interview survey of the general population in Japan, employing the time trade-off method.

The eligibility criteria for the survey were as follows.

[1] Inclusion criteria

- Those aged ≥20 years at the time of screening
- Those judged by the research investigators to have sufficient understanding of the conduct of the research study
- Those who consent to participate in this research study
- Those who can complete the procedures specified in the protocol
- Those living in Japan at the time of screening

[2] Exclusion criteria

- Those for whom obtaining consent or conducting the study is difficult due to insufficient Japanese language ability
- Those unable to participate in the survey themselves

The vignettes presented in the health state utility estimation using the time trade-off (TTO) method were developed per a literature search, clinical studies, and the opinions of clinical experts in PV. The vignettes comprised polycythemia vera symptoms, anxiety of second malignancies, and expectations of future treatment discontinuation or operational cure, and six vignettes were surveyed (Supplementary Table 1).

Responses were obtained via face-to-face questionnaires. The vignette-based TTO method questionnaire used a ping-pong titration method in which the maximum 10-year

survival was increased or decreased in 1-year increments. A pilot survey was conducted in advance with a small number of participants, and a cognitive debriefing of the questionnaire scenario was conducted to evaluate their understanding of the questionnaire scenarios before the main survey was conducted.

Statistical analysis

The utility for each health state was calculated per the results of a survey using the TTO method. For health states that were not considered to be "worse than death," the anchors were (0) death and (1) a completely healthy state, and calculations were per the following formula:

$$u = \frac{x}{y},$$

where x and y represent the respective durations of survival for each of the health states (as determined using the ping-pong titration method at the time point when the combination of the two health states and the duration of survival were judged to be equivalent by the respondents, or the mean durations of survival for each health state at the two respective time points when the superiority or inferiority of the two combinations was reversed).

The health state of "worse than death" was calculated per the following formula (in this instance, health state utility ranged from 0 to –1):

$$u' = \frac{x'}{10} - 1,$$

where x' denotes the mean duration of survival for only the completely healthy state (as determined via the ping-pong titration method at the time point when the combination of the two health states and the duration of survival were judged to be equivalent by the respondents, or the mean duration of survival if the respondent is only in the completely healthy state at the two time points when the superiority or inferiority of the two combinations was reversed).

Ethical approval

Prior to conducting the survey, the appropriateness and ethical considerations were reviewed by the Institutional Review Board of the Public Health Research Foundation (Approval number: 23L0005), and approval was obtained.

Supplementary Table 1. Vignettes used in the survey

	No improvement of PV symptoms /anxiety of second malignancies /no expectation of treatment discontinuation or operational cure	No improvement of PV symptoms /no anxiety of second malignancy /no expectation of treatment discontinuation or operational cure	No improvement of PV symptoms /no anxiety of second malignancy /expectation of treatment discontinuation or operational cure	Improvement of PV symptoms /anxiety of second malignancies /no expectation of treatment discontinuation or operational cure	Improvement of PV symptoms /no anxiety of second malignancy /no expectation of treatment discontinuation or operational cure	Improvement of PV symptoms /no anxiety of second malignancy /expectation of treatment discontinuation or operational cure
**Disease**	• Your bone marrow, which produces blood, is diseased	• Your bone marrow, which produces blood, is diseased	• Your bone marrow, which produces blood, is diseased	• Your bone marrow, which produces blood, is diseased	• Your bone marrow, which produces blood, is diseased	• Your bone marrow, which produces blood, is diseased
**Symptom**	• You may feel the need to rest immediately after any movement, and you may become tired quickly<br>• While sleeping, you may sweat so much that you have to change your underwear, pajamas, or sheets<br>• You may experience itching, which may get worse when you take a bath	• You may feel the need to rest immediately after any movement, and you may become tired quickly<br>• While sleeping, you may sweat so much that you have to change your underwear, pajamas, or sheets<br>• You may experience itching, which may get worse when you take a bath	• You may feel the need to rest immediately after any movement, and you may become tired quickly<br>• While sleeping, you may sweat so much that you have to change your underwear, pajamas, or sheets<br>• You may experience itching, which may get worse when you take a bath	• You may not feel the need to rest immediately after any movement, and you may not become tired quickly<br>• While sleeping, you may not sweat so much that you have to change your underwear, pajamas, or sheets<br>• You may not experience itching, which may get worse when you take a bath	• You may not feel the need to rest immediately after any movement, and you may not become tired quickly<br>• While sleeping, you may not sweat so much that you have to change your underwear, pajamas, or sheets<br>• You may not experience itching, which may get worse when you take a bath	• You may not feel the need to rest immediately after any movement, and you may not become tired quickly<br>• While sleeping, you may not sweat so much that you have to change your underwear, pajamas, or sheets<br>• You may not experience itching, which may get worse when you take a bath
**Anxiety of the future**	• I know that my blood vessels can become clogged more easily, which can lead to	• I know that my blood vessels can become clogged more easily, which can lead to	• I know that my blood vessels can become clogged more easily, which can lead to	• I know that my blood vessels becoming clogged more easily could lead to diseases	• I know that my blood vessels becoming clogged more easily could lead to diseases	• I know that my blood vessels becoming clogged more easily could lead to diseases

	diseases such as myocardial infarction and stroke, or even leukemia<br>• I know that the medication I am taking can cause cancer	diseases such as myocardial infarction and stroke, or even leukemia	diseases such as myocardial infarction and stroke, or even leukemia	such as myocardial infarction and stroke, or even leukemia, but the medications I am taking reduce the likelihood of this happening<br>• I know that the medication I am taking can cause cancer	such as myocardial infarction and stroke, or even leukemia, but the medications I am taking reduce the likelihood of this happening	such as myocardial infarction and stroke, or even leukemia, but the medications I am taking reduce the likelihood of this happening
**Hope of treatment discontinuation or operational cure**	• My disease will not go away for as long as I live, and I need to take medications on a regular basis for the rest of my life	• My disease will not go away for as long as I live, and I need to take medications on a regular basis for the rest of my life	• There is hope that, in the future, I may be able to stop treatment or be cured of the disease	• My disease will not go away for as long as I live, and I need to take medications on a regular basis for the rest of my life	• My disease will not go away for as long as I live, and I need to take medications on a regular basis for the rest of my life	• There is hope that, in the future, I may be able to stop treatment or be cured of the disease

PV, polycythemia vera.

Results

An interview survey was conducted for 208 participants (mean age: 44.5 years, 50.0% male). Informed consent was obtained from all individual participants included in the study. The mean health state utility (standard deviation [SD]) for "improvement of polycythemia vera symptoms/no anxiety of second malignancy/no expectation of treatment discontinuation or operational cure" was 0.776 (0.257). The mean health state utility (SD) for "improvement of polycythemia vera symptoms/no anxiety of second malignancy/expectation of treatment discontinuation or operational cure" was 0.810 (0.200), and the mean health state utility (SD) for "improvement of polycythemia vera symptoms/anxiety of second malignancies/no expectation of treatment discontinuation or operational cure" was 0.705 (0.294) (Supplementary Table 2).

Supplementary Table 2. Results of health state utility estimation using the TTO method

	**Respondents (n=208)**
Age, mean (SD), years	44.5 (13.93)
Female (%)	104 (50.0%)
Health state utility, mean (SD)	
No improvement of PV symptoms/anxiety of second malignancies/no expectation of treatment discontinuation or operational cure	0.416 (0.435)
No improvement of PV symptoms/no anxiety of second malignancy /no expectation of treatment discontinuation or operational cure	0.415 (0.481)
No improvement of PV symptoms/no anxiety of second malignancy/expectation of treatment discontinuation or operational cure	0.605 (0.360)
Improvement of PV symptoms/anxiety of second malignancies/no expectation of treatment discontinuation or operational cure	0.705 (0.294)
Improvement of PV symptoms/no anxiety of second malignancy/no expectation of treatment discontinuation or operational cure	0.776 (0.257)
Improvement of PV symptoms/no anxiety of second malignancy/expectation of treatment discontinuation or operational cure	0.810 (0.200)

PV, polycythemia vera; TTO, time trade-off.

Supplementary 2. Threshold analysis for price of ropeginterferon alfa-2b

Supplementary Figure 1. Threshold analysis for price of ropegIFN

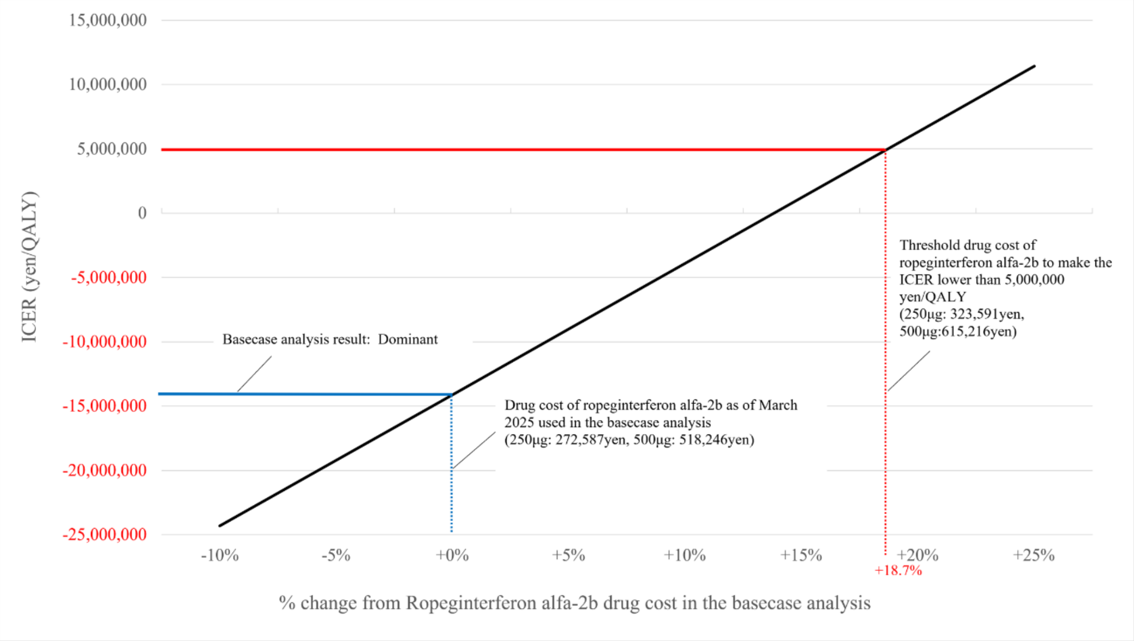

ICER, incremental cost-effectiveness ratio; RopegIFN, ropeginterferon alfa-2b, QALY, quality-adjusted life years.
